# Developing a web-based toolkit for new mothers about postpartum pelvic floor health in collaboration with a professional medical association

**DOI:** 10.5195/jmla.2021.1078

**Published:** 2021-10-01

**Authors:** Brandon Patterson, Lauren Clark, Ana C. Sanchez-Birkhead, Liliana I. Martinez, Marlene J. Egger

**Affiliations:** 1 b.patterson@utah.edu, Technology Engagement Librarian, Eccles Health Sciences Library, University of Utah, Salt Lake City, UT; 2 lclark@sonnet.ucla.edu, Professor, Shapiro Family Endowed Chair in Developmental Disabilities Studies, School of Nursing, UCLA, Los Angeles, CA; 3 ana.sanchez-birkhead@nurs.utah.edu, Associate Professor, College of Nursing, University of Utah, Salt Lake City, UT; 4 liliana.martinez@hci.utah.edu, Community Health Educator, Huntsman Cancer Institute, Salt Lake City, UT; 5 marlene.egger@hsc.utah.edu, Professor, Division of Public Health, Department of Family and Preventive Medicine, University of Utah, Salt Lake City, UT

**Keywords:** toolkit, Internet

## Abstract

**Background:**

Few electronic resources are available for new mothers with concerns about changes in their pelvic floor following childbirth. Patients may struggle when seeking authoritative information regarding pelvic floor conditions online given the sensitivity of the topic as well as the inadvertent connection to obscene or demeaning content found online. A health sciences librarian partnered with the Motherhood and Pelvic Health Study, an interdisciplinary research group, to provide expert searching skills for a particularly challenging health condition that patients struggle to find useful information on.

**Case Presentation:**

A custom rubric was developed to evaluate existing information products, which included criteria for cultural sensitivity, conflicts of interest, and other red flags. This evaluation process enabled the research team to identify top-tier evidence-based materials that were culturally congruent. This collaborative evaluation process led to the creation of a web-based toolkit resource for new mothers concerned about changes in their pelvic floor. The toolkit connects women to pertinent information on a national health organization's patient portal, supplemented by videos created by the team to serve as models of communication for women and health care providers.

**Conclusion:**

When developing a web-based resource, health sciences libraries can partner with research teams to find, evaluate, and disseminate information. Culturally congruent toolkits such as this one can improve access to health information and lead to improved health outcomes. To ensure that the information highlighted in toolkits is both culturally congruent and authoritative, research teams should form advisory committees and partner with relevant professional medical associations.

## BACKGROUND

Patients advance their own health through self-advocacy, and toolkits pave the way for successful and informed care partnerships with providers. Health care providers also use toolkits, fact sheets, and other information to improve patient access to health education and resources for populations with limited health literacy [1]. The Agency for Healthcare Research and Quality (AHRQ) defines toolkits as “a collection of related information, resources, or tools that together can guide users to develop a plan or organize efforts to follow evidence-based recommendations or meet evidence-based specific standards” [2]. Toolkits can help patients and care providers understand and retain health care information by avoiding medical jargon, limiting information to two or three important points at a time, and offering examples in videotaped simulated patient sessions [3]. We followed AHRQ guidance for presenting action-oriented recommendations following a standard format, which addresses organization, design, and language needs; this format was intended to be user centered [4].

This study stemmed from the Motherhood and Pelvic Health (MAP) Study funded by the Eunice Kennedy Shriver National Institute of Child Health and Human Development [5]. A multiple methods study, MAP investigated the effects of physical activity, cultural context, and the biomechanical forces involved in childbirth on women's pelvic floor health in their first year after a vaginal delivery. The study aimed to develop translational resources for Mexican American and Euro-American women, in partnership with a community advisory committee consisting of older women who were knowledgeable about pelvic floor disorders and new mothers with limited experiences related to changes in their pelvic floor [6]. The women were split into two groups out of respect for their language and cultural differences, so that one group spoke English while the second group spoke mostly Spanish. The women had varied educational backgrounds. Three sessions of the advisory committee meetings were structured as focus groups, consisting of eight or fewer participants who had completed signed consent forms and agreed to ground rules for confidentiality. Each meeting proceeded with a flexible moderator's guide of topics, with one to two researchers of cognate ethnicity present; the full research team later reviewed notes and transcripts of the meetings.

This study sought to incorporate the experiences of these focus group participants in the selection and development of resources. We found that women in focus group discussions might call the changes they experienced one term, which was different than what they would look up online, which was different than what was used at doctor visits. For example, in several sessions, women described the pelvic floor symptoms they were having as a “blockage” or “bolita” or “leaking,” found it difficult to search the Internet about such search terms, and then found that doctors would use “bulge” and “incontinence.” It turned out that many of the women received information through friends and family members, relying on informal channels of communication. Women were generally cautious about involving a doctor in a discussion of symptoms that were challenging to explain across a chasm of vocabulary differences.

## CASE PRESENTATION

This case study presents the work of an interdisciplinary team comprised of a health sciences librarian and faculty from nursing, medicine, bioengineering, and public health who created a toolkit concerning women's postpartum pelvic floor health. We discuss ways academic librarians can assist teams to find relevant evidence and evaluative frameworks to support discipline-specific educational resources. We also suggest ways an advisory committee and a professional medical association strengthen the final product via authentic review from patients and clinicians. The result launched a web-based toolkit for new mothers, produced by a team of faculty, staff, and community members in collaboration with the American Urogynecologic Society (AUGS). The toolkit provides new mothers with personal narratives to normalize the range of postpartum experiences, connects them to evidence-based resources about pelvic floor support changes postpartum, and incorporates cultural viewpoints and language that feel inviting and comfortable for our focal populations, specifically Mexican American and Euro-American women [6]. Pelvic organ prolapse is generally diagnosed in older women, so new mothers with less severe pelvic floor changes often struggle with finding appropriate resources according to group responses documented.

For toolkit development, we relied on a guide by Garcia et. al. to compile twenty of the top resources for women experiencing prolapse [7]. Resources in the toolkit have been compiled and can be viewed along with videos that we developed for the AUGS Voices for Pelvic Floor Disorder (PFD) website [8]. The librarian found information products and provided expertise to find, evaluate, and disseminate sources of information. To form a cohesive and effective resource, we created a rigorous review process to evaluate information for the toolkit. We outline our process to develop a strategy for the toolkit in four steps listed below.

### Step 1: Design and decision-making about toolkit resources

To critically evaluate existing materials, the team determined the resource appraisal protocol. The librarian supported the process on decision-making by providing ready-made strategies for searching, evaluating, and disseminating information. Multiple stakeholders participated in shaping the appraisal strategies. Community advisory committee members provided recommendations for the planned resource, feedback about our focused ethnography, and suggestions for developing the tools we curated and produced. To enhance the availability and longevity of the toolkit, we partnered with a professional medical association, AUGS, to provide a venue for the information to be shared.

We determined most resources included medical terms that were of average difficulty to read, so to align with existing information found on the AUGS website, we included resources that were at a seventh-grade reading level [9]. We prioritized resources in four categories recommended by our advisory committee. These include the following:

- Credible consumer resources (e.g., patient education materials, resources produced by advocacy or consumer health groups)
- Multimedia resources (e.g., video, graphic novels, podcasts)
- First-person cultural narratives and resources that provide accounts of the postpartum pelvic floor changes and the experience of those changes and pathways to care (e.g., blogs)
- Professional connections to access resources for additional help (e.g. American College of Obstetricians and Gynecologists, AUGS, American College of Nurse-Midwives, and other professional connections and sources)

### Step 2: Searching for relevant resources

To begin our search strategy, we identified records through popular search engines and databases. We utilized consumer search engines (e.g., Google, Amazon, etc.) to identify web-based information, books, and products available to consumers, as well as PubMed and MedlinePlus to search for medical literature and health information. We removed duplicate items and then screened records to ensure they fit within our protocol. We supplemented our search by asking colleagues and community advisory committee members to share resources and links that they found useful.

### Step 3: Rating resources for quality and fit

The relevance and value of resources varied, so we selected formalized criteria to evaluate information resources for the toolkit. We used a scoring key adapted from Garcia et al., where point values were given to specific resources based on their alignment with the CRAAP test (Supplemental Table 1) [7]. Other similar assessment tools of online content for health information (e.g. DISCERN, MERSQI) were considered but not used because the population of focus consisted mostly of novice information seekers, and the sources were not limited to medical or patient education material. The CRAAP test, which has gained widespread adoption since its debut in 2004, consists of twenty-five questions organized into five domains: currency, relevancy, authority, accuracy, and purpose [10–11].

As noted by Garcia et al., the five domains of the CRAAP test are limited in how they represent diverse patient populations in the US and online content in other languages [7]. To address this limitation, we used clinical expertise on navigating culture and meaning in health services research to add to the existing scoring key [6, 12]. Aware of the sensitivity behind searching keywords synonymous with pelvic floor health, we included additional protective measures that could catch potentially harmful content. Thus, three additional domains were included in our rubric: cultural sensitivity (four items), conflict of interest (one item), and red flags (one item) (Supplemental Table 2).

In order to identify and elevate *culturally-sensitive* resources on pelvic floor disorders, criteria on the topic were developed to supplement the existing CRAAP test score. We established four items within the category that came from literature on choosing culturally appropriate resources [13–17]. The items we established are listed below:

- Content: The information provided is accurate and respectful of cultural beliefs, values, and attitudes. The stories told are positive and respectful of the cultures of women.
- Audience: The material is developed for Mexican American and Euro-American women.
- Language: The information is provided in English and/or Spanish and conveys respect for Mexican American and Euro-American women and their traditions, beliefs, and values.
- Visual images: The material contains images that reflect experiences of Mexican American and/or Euro-America women. The images complement the information provided.

*Conflict of interest* criteria were established through conversations with the community advisory committee who often preferred resources coming from first-person accounts or testimonials but wanted a way to verify that an author of the resource was credible. For the purpose of this toolkit, we defined conflict of interest to mean that the primary objective of the resource may be influenced or compromised by private or personal interest. This includes financial interest, promoting religion, politics, and products, or services sold for profit. To preserve the integrity of the scoring system that rated resources discussing a sensitive topic while also addressing any prevalent sources that could offend our intended audience, we established a term titled *red flags*. This term was designed to be invoked if the research team thought a nominated site contained graphic or obscene content. Red flags were used as a catch-all category; when selected during the scoring process, a red flag would signal removal from the toolkit. Red-flagged resources included resources whose authors profited from endorsement of materials or aids therein, resources that used profanity, resources that featured images that could be construed as semipornographic or demeaning to patients, as well as resources that included disparaging remarks about health care providers.

After scoring resources from each priority category, the top choices were identified for use in the toolkit. Top choice resources included devices or products approved by the US Food and Drug Administration (FDA), exercises recommended by clinicians and therapists for strengthening the pelvic floor (Kegels, abdominals, lower back, hip muscle exercises), and professionally validated self-screening instruments. Any resources that had insufficient content, were debunked in the scientific literature, pertained specifically to men, or were endorsed by celebrities but lacked FDA approval were excluded from our scoring key. Both the total score sum and average score were calculated, with a higher score indicating a better-quality resource. Notes and discussions were conducted for resources that had identical total sum scores within each category to determine the top resource.

### Step 4: Packaging and distributing resources

The research team consulted with representatives from the medical organization to help with the design, layout, and interactions of the website. Our design took several iterations with the initial concept partially inspired by a website created by the librarian team member at a prior institution [18]. There were trade-offs in design compatibility with the AUGS website, such as its web page color scheme versus color schemes that would have been more traditionally Mexican American. After agreeing on a look and format that would be compatible with the AUGS website platform, we created flowcharts for each webpage where content, available in both English and Spanish, could be pulled from to create the web resource. Our resource toolkit is now published as the New Moms tab of the AUGS Voices for PFD website, including the list of curated resources, customizable menu items using terms used by women, and videos created by the team. We have a brochure referencing the website via QR code that can be handed out or posted on a variety of related sites to direct women to this resource.

## DISCUSSION

As experts in information, literature searching, evidence synthesis, and the publication process, academic librarians are well-equipped to collaborate with developers of electronic resources to design resource evaluation processes that can lead to better health information toolkits that meet the needs of patients and health care professionals. While toolkits have become a more prevalent means of sharing health information resources, it is important that they also serve a diverse set of information seekers. Advisory committees can play a crucial role in this process, selecting resources that are credible and culturally sensitive. During sessions, our advisory committee participants answered questions about what they would like to see in the toolkit, preferences in screening materials to use in the toolkit, and whether they found the resources helpful. This proved to be a valuable process that will be used in future resource-driven projects.

Upon completion of the toolkit, several women from the community advisory group reported their appreciation of the effort put toward it, and a few older women commented that they wished they would have had the resource as a new mother. Although no additional follow-up has been scheduled, we would be interested in feedback from women who used the toolkit, regarding how useful they found it, whether they found the material culturally sensitive, and if they would tell their friends about the resource. Updates to the toolkit will be at the discretion of the AUGS organization. Future research can provide better understanding of the vernacular used by patients seeking pelvic floor health information and the cultural issues that help inform our understanding of the complicated context-related factors facilitating or barring successful adoption of the toolkit.

Throughout this process, we gained a better understanding of the limitations of online information seeking, especially when biomedical terminology varies greatly from lay audience vernacular. Scoring keys can help those making a toolkit provide resources that are independently evaluated for relevancy and authority, while also being culturally sensitive. Collaborating with a well-established medical organization can help to build bridges of vocabulary, to incorporate professional review, and to legitimize the resource being offered. By condensing and disseminating information in this way, accurate health information becomes more accessible and ultimately can improve health outcomes.

## ASSOCIATED DATA

The Motherhood and Pelvic Health Study Group Scoring Key and data collected associated with this article is available in the Open Science Framework at https://osf.io/asbcz/.

## SUPPLEMENTAL FILES

**Supplemental Table 1.** CRAAP Test and scoring key

**Supplemental Table 2.** Additional Scoring Key
