# Supplementary material for: Developing a web-based toolkit for new mothers about postpartum pelvic floor health in collaboration with a professional medical association
Source: J Med Libr Assoc. 2021 Oct 1;109(4):667–71. doi: 10.5195/jmla.2021.1078 (PMC8608176; doi:10.5195/jmla.2021.1078)
Supplement: Supplementary file 1 — Supplemental Table 1. CRAAP Test and scoring key [file jmla-109-4-667-s01.docx]

Supplemental Table 1. CRAAP Test and scoring key

|  |  | | | |
| --- | --- | --- | --- | --- |
|  | 0 | 1 | 2 | 3 |
| Currency (3 Items, up to 4 points) |  |  |  |  |
| Date created | >5 years | 1–5 years | <1 year |  |
| Info updated | No | Yes |  |  |
| Embedded links functional | No | Yes |  |  |
| Relevance (4 items, up to 5 points) |  |  |  |  |
| Answers questions appropriately | No | Yes |  |  |
| Intended audience | Not relevant | Health care provider | Patient/family |  |
| Info appropriate level for needs | No | Yes |  |  |
| Comfortable citing source | No | Yes |  |  |
| Authority (5 Items, up to 9 points) |  |  |  |  |
| Identity of author | None identified | Patient | Group connected to topic | Authoritative resource |
| Author credentials | None identified | Patient | Group connected to topic | Authoritative resource |
| Author qualified to write on topic | No | Yes |  |  |
| Contact info of author provided | No | Yes |  |  |
| URL identifies something relevant | No | Yes |  |  |
| Accuracy (6 Items, up to 8 points) |  |  |  |  |
| Derivation of information | Unclear | Personal experience | Professional experience | Evidence-based review |
| Information supported by evidence | No | Yes |  |  |
| Information reviewed or referred | No | Yes |  |  |
| Information verified by 2nd source | No | Yes |  |  |
| Information unbiased, unemotional | No | Yes |  |  |
| Grammar errors & typos | Yes | No |  |  |
| Purpose (5 Items, up to 8 points) |  |  |  |  |
| Purpose of information | Selling something | Persuade or entertain | Inform | Teach |
| Intentions or purpose is clear | No | Yes |  |  |
| Nature of info | Propaganda | Opinion | Facts |  |
| POV appears objective & impartial | No | Yes |  |  |
| Political, ideological, cultural, or  religious biases | Yes | No |  |  |
