# Supplementary material for: Developing a web-based toolkit for new mothers about postpartum pelvic floor health in collaboration with a professional medical association
Source: J Med Libr Assoc. 2021 Oct 1;109(4):667–71. doi: 10.5195/jmla.2021.1078 (PMC8608176; doi:10.5195/jmla.2021.1078)
Supplement: Supplementary file 2 — Supplemental Table 2. Additional Scoring Key [file jmla-109-4-667-s02.docx]

Supplemental Table 2. Additional Scoring Key

|  |  | | | |
| --- | --- | --- | --- | --- |
|  | 0 | 1 | 2 | 3 |
| Cultural sensitivity  (4 Items, up to 8 points) |  |  |  |  |
| Content | No | Neutral | Yes |  |
| Audience | Not relevant | Women (EA and/or MA) | Patient & family |  |
| Language | Only in one language and/or NOT respectful of traditions, beliefs, and values | Yes, language of material respects EA/MA, 1 language | More than one language & language of the material is respectful of EA/MA traditions, beliefs, and values |  |
| Visual | No image/image doesn’t support information | Image supports information | Positive images reflect EA and/or MA cultures |  |
| Conflict of interest  (1 item, up to 1 point) |  |  |  |  |
| Includes:   • Financial interest  • Promoting religion  • Promoting politics • Promoting products  • Promoting services | Yes | No |  |  |
| Red flag  (invalidated resource) |  |  |  |  |
| Includes:  • Inappropriate  • Swearing  • Semipornographic pictures  • Insult leveled at women or men  • Disparaging remarks about health care providers | Yes | No |  |  |
